# Toward an Improved Understanding of the Role of Dielectrics in Capacitors

**DOI:** 10.3390/ma11091519

**Published:** 2018-08-24

**Authors:** Jonathan Phillips

**Affiliations:** Energy Academic Group, Naval Postgraduate School, Monterey, CA 93943, USA; jphillip@nps.edu

**Keywords:** capacitor, super dielectric material, dielectric theory

## Abstract

A new fundamental principle of the theory of dielectrics in capacitors is demonstrated. That is, dielectric material in any geometry that reduces the field generated by charges on capacitor electrodes is effective in increasing capacitance. Specifically, it is shown that super dielectric material on the outer surfaces of the electrodes of a parallel plate capacitor increases dielectric constant, as well as energy and power densities, by orders of magnitude. The implicit assumption in all current capacitor theory, that the “capacitor” is only that region occupied by the electrodes and the space between them, is shown to be incorrect.

## 1. Introduction

This study was designed to test a natural extension of the super dielectric material (SDM) model, recently advanced elsewhere [1,2,3], regarding dielectric material. The model can be reduced to the following two principles: (i) The field at all points in space generated by dielectric material associated with a capacitor determines net effective dielectric constant, and (ii) The fields of a dielectric are proportional to the length and densities of dipoles within the dielectric, which are induced by charge on the electrodes. This model can be understood by contrasting it with several implicit aspects of the standard, or ‘”text book” hypothesis of dielectric behavior [4,5,6,7]. (i) Implicit in the standard explanation of the action of dielectrics in parallel plate capacitors is the following: only the electric field between the electrodes is impacted by the dielectric. In contrast, the SDM postulate advanced above indicates that the field at all points in space, not just between electrodes, is impacted by the dielectric and in turn the field beyond the electrodes impacts the effective dielectric constant. (ii) The standard hypothesis implies that any material, whatever the dielectric value of that material, outside the volume between the electrodes will have no impact on the effective dielectric constant. In contrast, the SDM model leads naturally to a remarkable postulate regarding the impact of dielectric material outside the volume between the electrodes. Specifically, implicit in the SDM model is the postulate that dielectric material outside the volume between the electrodes, that is, all “associated” dielectric material, both between the plates and nearby dielectric material, can impact the “effective” dielectric constant. Just as the dielectric material between the electrodes can impact the electric field at all points in space, so can dielectric material outside this region impact the electric field at all points in space, including the volume between the electrodes.

A simple test of the postulate was conducted. A form of super dielectric material (SDM), a recently invented class of materials [1,2,3,8,9,10,11,12,13,14,15,16], was placed on the outside of several parallel plate capacitors. In all cases the core structure, classically considered “the capacitor”, consisted of two titanium metal electrodes with a thin sheet of low dielectric constant (<200) material between. The SDM on the outside of the capacitor dramatically increased the effective dielectric constant below ~1.2 V in all cases. In several cases, the observed effective dielectric constant was more than 10^6^ times of that measured for the core structure.

A second objective of this study was to demonstrate that SDM can be observed with metal electrodes as well as the carbon electrodes employed in all previous studies [1,2,3,8,9,10,11,12,13,14,15,16]. Titanium was selected as the electrode material, as more traditional electrode materials, such as copper and aluminum were found to corrode rapidly due to the aggressive chemistry generated by SDM at elevated voltage. Using titanium electrodes, and an SDM gel created from fumed silica mixed with an aqueous NaCl solution, dielectric values, below ~1.2 V, as high as 5 × 10^9^ were observed.

## 2. Experimental Method

**Figure 1:**
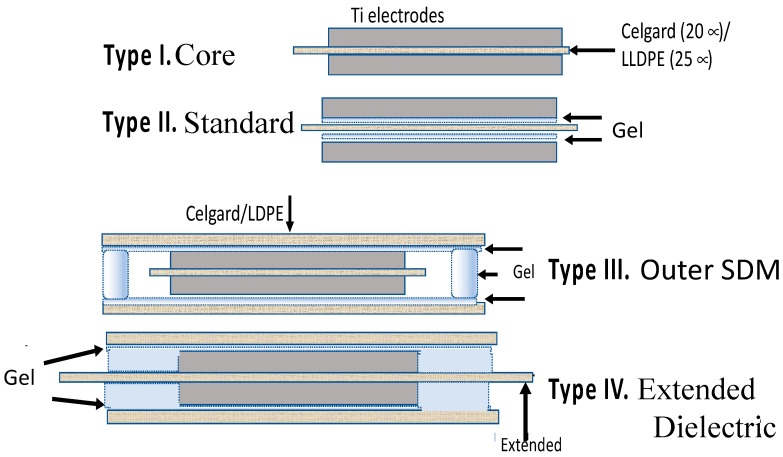
The four capacitor geometries studied. These geometries were selected to compare/contrast two models. For example, the standard paradigm indicates Type III should perform exactly as Type I, whereas super dielectric material (SDM) theory predicts Type III will have far higher dielectric constant, energy density, etc. than Type I. Type IV is equivalent to an electric double layer capacitors (EDLC)/supercapacitor configuration in the sense the two “electrodes” are separated by an electrically insulating divider. In fact, Celgard is often used in that role in EDLC.

Four capacitor geometries (Figure 1) were studied using a constant current galvanostatic approach.

Type I: Core—The Type I: Core capacitors were constructed following the normal paradigm for parallel plate capacitors, that is, a non-electrically conducting material (dielectric) is placed between two conductive electrodes. The specific capacitor configuration employed 2 cm × 2 cm × 0.01 mm titanium metal electrodes, each with a 3 cm × 3 mm × 0.1 mm tail/contact, and a dielectric made of either a 2.5 cm × 2.5 cm × 20 micron square of a microporous material generally employed as a separator between anode and cathode in batteries and capacitors, Celgard 2320, or a 2.5 cm × 2.5 cm × 25 micron square of polyethylene-linear low density (LLDPE). The dielectric was placed between the electrodes such that it extended in all directions slightly beyond the area enclosed by the titanium metal electrodes.

Type II: Standard—The primary difference between the Type II: Standard construction and Type I: Core is the addition of an effective, viscous, SDM gel, described elsewhere [10,16] and in the next section. Both sides of the dielectric sheet were coated with a thin layer of the gel, as shown.

Type III: Outer SDM—A capacitor configuration, unique to the present study, Type III: Outer SDM, consists of a core identical to the Type I: Core capacitor, but with layers of SDM affixed to the outside surfaces of both metal electrodes. The SDM gel on the outside surfaces is organized such that all the super dielectric material was in electric contact. Specifically, two 3.5 cm × 3.5 cm × 20 micron sections of Celgard 2320, or 25 micron thick LLDPE of the same lateral dimensions, were coated on one side with super dielectric gel, a different thickness in each of repeat experiments (Table 1). One coated sheet was placed on top of each electrode, that is, outside the volume generally considered to be the capacitor. For both electrodes the coated sheet was placed such that dielectric gel was in direct contact with the electrode. As these outer sheets were larger than the dielectric sheet, the super dielectric gel from the top gel coated sheet was in direct contact with the dielectric gel of the bottom electrode, and the entire Type I: Core enclosed in a continuous layer of SDM.

Type IV: Extended—This novel capacitor configuration, Type IV: Extended, starts with a modified Type I: Core. The modification was to enlarge the central dielectric sheet to a final size of 5 cm × 5 cm. Next, similar to the Type III: Outer SDM capacitor, a layer of super dielectric material was affixed to the outside metal electrode surfaces by using gel coated 3.5 cm × 3.5 cm Celgard or LLDPE. Unlike the Type III capacitors, the outer surface super dielectric gels were organized such that the super dielectric gel on the top and bottom electrodes were not in physical/electrical contact. Specifically, as the center dielectric material was a larger square than the top and bottom gel coated polymers, the gel on top and the gel on the bottom were not in contact.

Super Dielectric Material—In this case, as in earlier studies [1,10,11,16], a gel composed of fumed silica (Sigma Aldrich, 0.007 μm avg. particle size, St. Louis, MO, USA), and water with dissolved NaCl (Sigma Aldrich 10 mesh anhydrous beads) were employed. The weight ratio employed Silica/NaCl/H_2_O was 1/2.3/8.7. This fumed silica is a very hygroscopic material, which reaches the point of “incipient wetness” at a weight ratio of 8.8 parts water to 1 part fumed silica, similar to that discussed elsewhere [10]. Clearly, the gel is at least 80% water by volume, allowing ions to travel virtually unimpeded through the entire gel. The salt weight reflects a weight concentration of about 25% in water, safely below the saturation concentration of NaCl in water at 298 K, ~36%. The gel formed is a nearly transparent/white color, very viscous, and holds whatever shape it is molded into for prolonged periods. The gel shows no sign of “water leakage”, even when placed on an adsorbent material.

**Figure 2:**
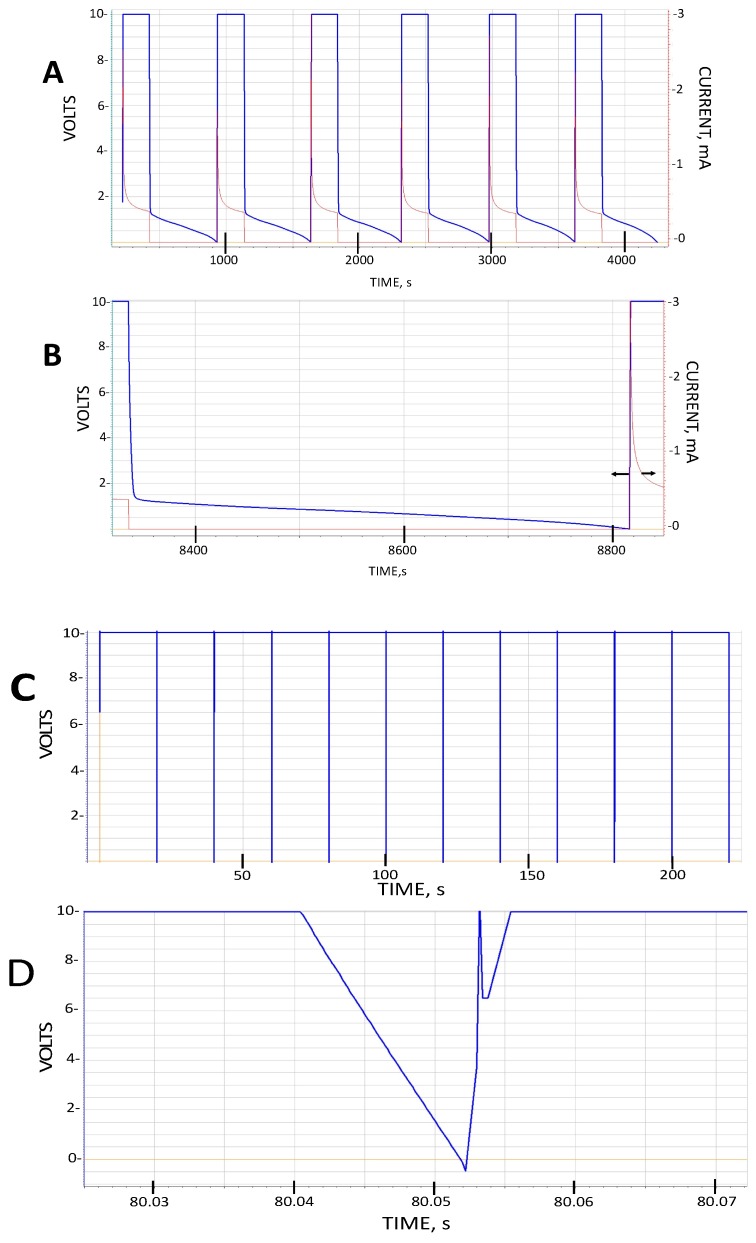
Exemplary data. (**A**) Six cycles, discharge current 0.05 mA from Type III capacitor built with LLDPE. (**B**) Single cycle, discharge current 0.05 mA, from Type III capacitor built with Celgard. (**C**) Eleven cycles, discharge current 0.05 mA, from Type I built with Celgard. (**D**) One cycle enlarged from (**C**). Note that in the constant current mode the galvanostat overshoots the minimum voltage and maximum voltage (U-shaped region is the instrument self-correcting). Also, Discharge Time(B)/Discharge Time(D) = 500,000 below one volt. These capacitors are identical except for dielectric gel outside the volume, which according to the standard paradigm, constitutes the capacitor.

Testing Protocol—Dielectric constant, energy and power density, were computed from the constant current discharge leg of a charge/hold/discharge protocol programed into a galvanostat (BioLogic Model SP 300 Galvanostat, Bio-Logic Science Instruments SAS, Claix, France). The device was operated in constant current charge/discharge mode over the voltage range, 0 to 10 V. As noted elsewhere [1], data collected in this mode readily yields capacitance (current divided by the slope of the voltage-time data), which according to the standard model, in turn is readily converted to dielectric constant (*ε*) by Equation (1) for a parallel plate capacitor:

(1) $$\;\varepsilon =\frac{C\;\times \;t\;}{A\;\times \;\varepsilon_{0}}$$

where *C* is the measured capacitance, *t* is the thickness of the dielectric layer, *A* is the area of the electrode and *ε*_0_ is the permittivity of free space. The data collected using this method is far easier to deconvolute than alternative methods such as cyclic voltammetry [17,18]. Also, unlike impedance spectroscopy [19,20,21], which is limited to providing values based on measurements conducted over a very small voltage range, ±15 mV, the constant current method uses data collected over the full voltage range to determine energy and power. The only true independent variable, the value of the constant current, is adjusted to provide different discharge times, hence “frequency dependent” information. Indeed, the higher the current, the shorter the discharge time. Generally, the reported values of parameters are the average of 10 cycles (ca. Figure 2).

Equation (1) is based on the assumption that only the dielectric material between the electrodes contributes to the capacitance. In the present study, this is clearly demonstrated to be an incorrect assumption. This presents a challenge regarding how best to report the experimental results. As the dielectric material in Type I and Type II is only between the electrodes, the computed dielectric, energy density, etc. values do meet the standard definitions. For Types III and IV there is dielectric material outside the standard geometric bounds of a capacitor, thus the dielectric constant, energy density and power density reported must be considered “effective” values. For these types of capacitors values are computed as if the only volume of significance is that of the dielectric between the plates. Specifically, the volume employed in computation is that fraction of the Celgard sheet or LLDPE between the electrodes. For Type III and Type IV capacitors, the volume of the gel outside the volume between the plates is not considered in computing any capacitor values. Another related consideration is the determination of the thickness of the dielectric layer for computation of dielectric constant, etc. In all cases, the volume is that of the Celgard or LLDPE sheet, plus the volume of the gel. The latter is computed on the basis of the density of the gel (1.33 g/cm^3^) divided by the precisely determined weight of gel. It is assumed the gel is evenly spread. 

Absolute values of energy and power are computed without regard to geometry. Energy is computed as the integral of area under the voltage time data (volts × sec) multiplied by current (amps), and power is computed as the total energy of the discharge divided by the total discharge time.

It was found in this study that there are essentially two ranges of capacitance as a function of voltage clearly distinguishable during the discharge. The first range from 10 V to ~1.2 V is very low and not a subject of significant inquiry in this study. The second range, orders of magnitude higher, is from 1.2 V to 0 V. For this reason, the capacitance and dielectric values reported are only reported based on data for the discharge between ~1.0 and 0 V. In this voltage regime the voltage vs time relationship was always found to be nearly linear for all discharge times greater than ~1 s, indicating constant capacitance over this voltage region. Given the extreme dependence of capacitance on discharge time even at ca. 0.1 Hz, NP supercapacitors, like all other supercapacitors, are not appropriate for use in electronic systems. The proposed applications of these capacitors, energy storage or power are largely low frequency (ca. 1 Hz) processes [1].

It is also notable that the standard protocol for capacitance testing involved three steps. The first step, in all cases, was charging to 10 V at 3 mA. The second step, in all cases, was to hold the voltage at 10 V for 200 s. The final step, the one from which all parameters were derived, was to discharge the capacitor at a constant current. The value of the discharge current is the only parameter modified in this study. As discussed above, it was varied to provide a range of discharge times. Note: This three-step protocol is very similar to that employed to characterize the capacitance of commercial supercapacitors [22,23].

## 3. Results

The experiments were designed to collect capacitance, dielectric constant, energy and power density data in order to test/contrast two hypotheses regarding how the four types of capacitors should behave. The standard hypothesis clearly predicts Types I, III and IV should behave identically because the core capacitors in all cases, two electrodes with a thin section of low dielectric constant organic/polymeric “dielectric” between, are effectively identical. In contrast, SDM theory predicts Type I and Type III capacitors will show radically different behavior. For example, Type III is predicted to have a dielectric constant orders of magnitude higher than Type I. The data, presented below, is only consistent with the SDM hypothesis. It is also notable that SDM behavior in a system employing metal electrodes is reported herein for the first time.

Type I—As shown in Figure 2, even at the lowest stable constant current setting of the Galvanostat, the discharge following prolonged charging at 10 V for both Celgard and LLDPE dielectrics, is remarkably rapid, approximately (note uncertainty due to voltage overshoot) 0.01 s/10 V at a discharge current of 0.05 mA. This is consistent with a very low dielectric constant (Table 1 and Table 2), in the range anticipated for most polymeric materials. Extensive analysis of Type I behavior is not presented because as shown below, all values, capacitance, dielectric constant, energy and power density are very, very low compared to Type II, III and IV capacitors.

**Figure 3:**
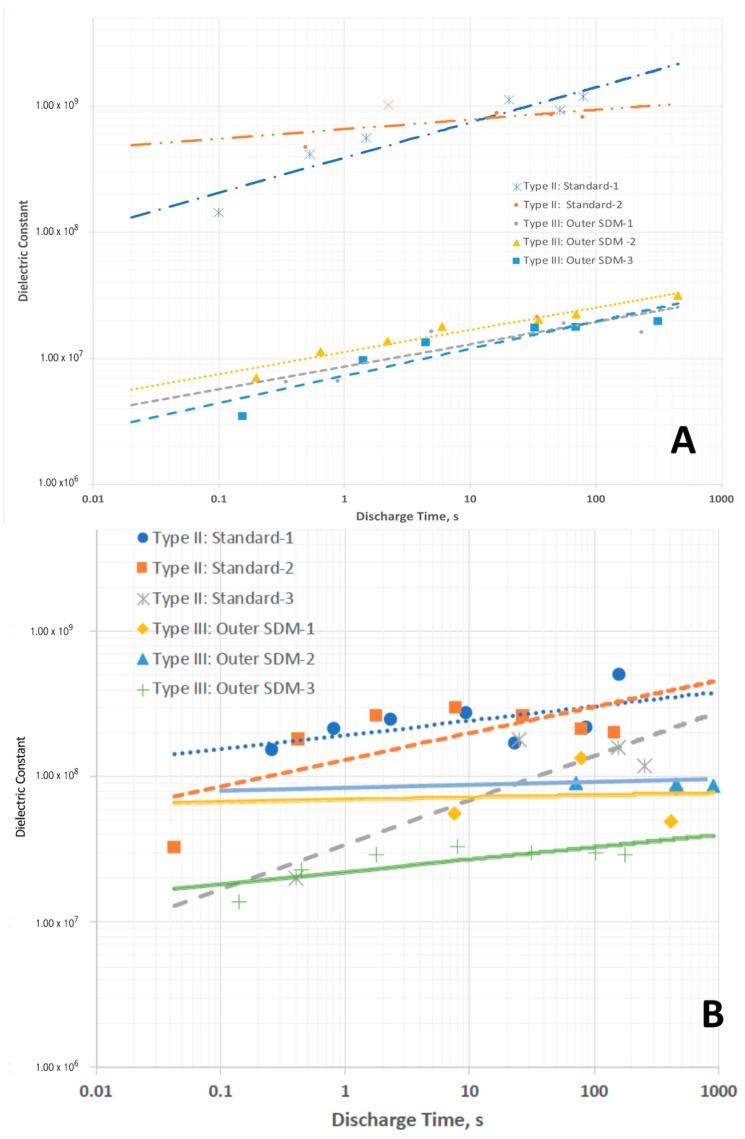
Effective dielectric constants, below ~1 V, of Type II and Type III capacitors. (**A**) Data from capacitors using Celgard dielectric, (**B**) Data from capacitors with LLDPE dielectric. All dashed lines are fits to Type II: Standard. All solid lines are fits to Type III: Outer SDM. Same general trends as for Celgard, but more scatter.

Type II—As shown in Figure 2, the discharge time for these capacitors, even at far higher discharge currents than employed to study Type I capacitors is orders of magnitude longer than Type I discharges. This translates, for voltages lower than about 1.2 V, into dielectric constants >10^7^ (Figure 3) even for very rapid discharges, a result only found for SDM. In particular, the magnitudes of the dielectric constants measured in the low voltage regime are very similar to those reported earlier for SDM-based capacitors (NP supercapacitors) built with a gel of a very similar composition, employing carbon electrodes, and also only below ~1 V. Another notable aspect of the observations here, and in all other SDM studies, is that the dielectric constant decreases as the discharge time is decreased. In fact, roll-off of capacitance with increasing frequency/decreased discharge time is anticipated for capacitors of all types.

The restricted voltage for valid dielectric value measurements is consistent with observations made in earlier studies with NP supercapacitors. In all cases high capacitance and dielectric values are only observed below ~1 V, as per the present case. Above this voltage the dielectric constant for Type II capacitors is very low and not a subject of the present investigation.

It is also clear that dielectric values as a function of discharge time for Type II capacitors are not perfectly fit by power law expressions, particularly those built with LLDPE. This is consistent with earlier observations that dielectric values, although indicative of all behaviors for ceramic and other types of capacitors, are not the best indicator of performance of any type of supercapacitor including NP supercapacitors. One difficulty with the use of this parameter for the NP supercapacitor is that of the method employed to determine it. As the capacitance is clearly a function of voltage, so is the dielectric constant. There is no absolute voltage below which the capacitance is constant, thus some error in the selection of voltage range leads, inevitably, to uncertainty in the reported value.

**Figure 4:**
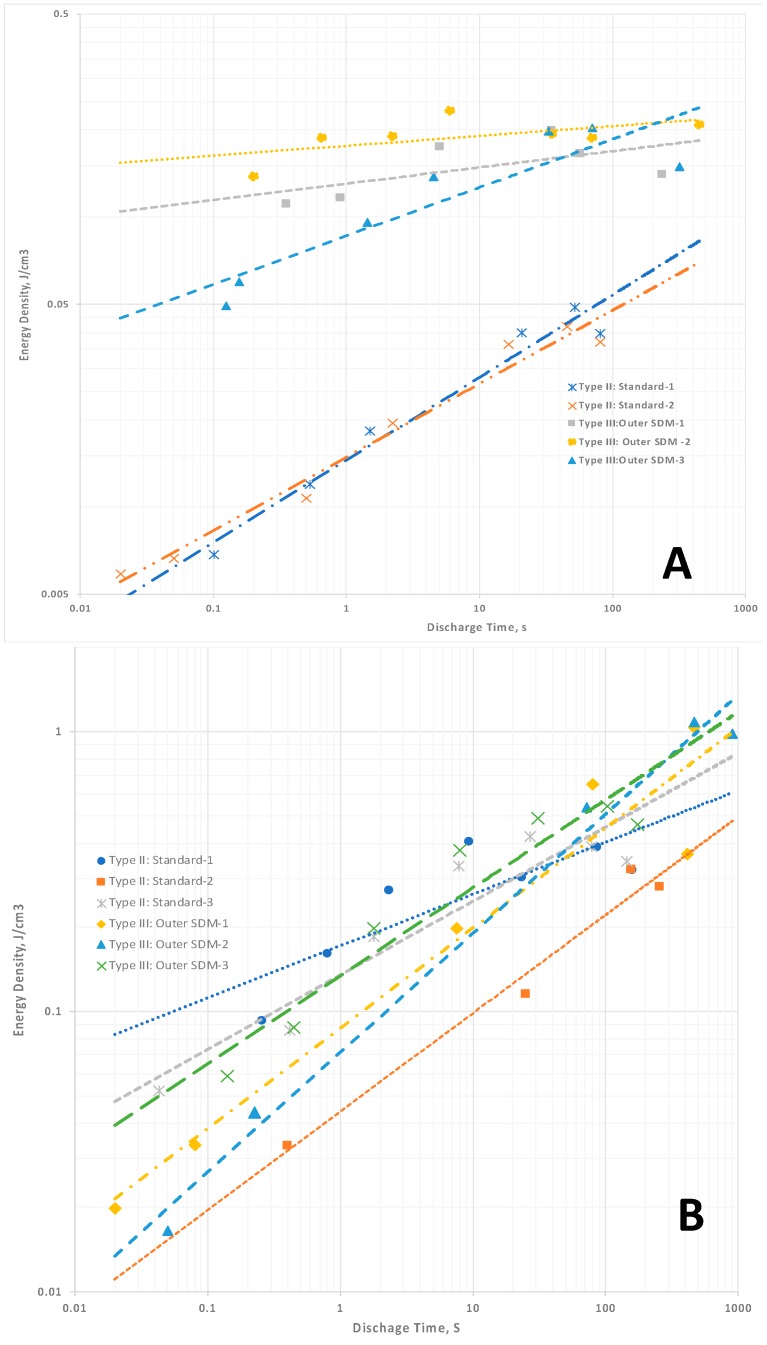
Energy density. (**A**) Celgard-based capacitors. The energy density for the Type II capacitors follows a clear power law function, whereas the Type III capacitor behavior is more chaotic. (**B**) LLDPE-based capacitors. There is a monotonic drop in energy density with decreasing discharge time, but this is not well fitted with a power law. Note: The values for energy density computed for Type III are arguably inflated because of the assumption energy is only associated with the Type I: Core dielectric volume.

**Figure 5:**
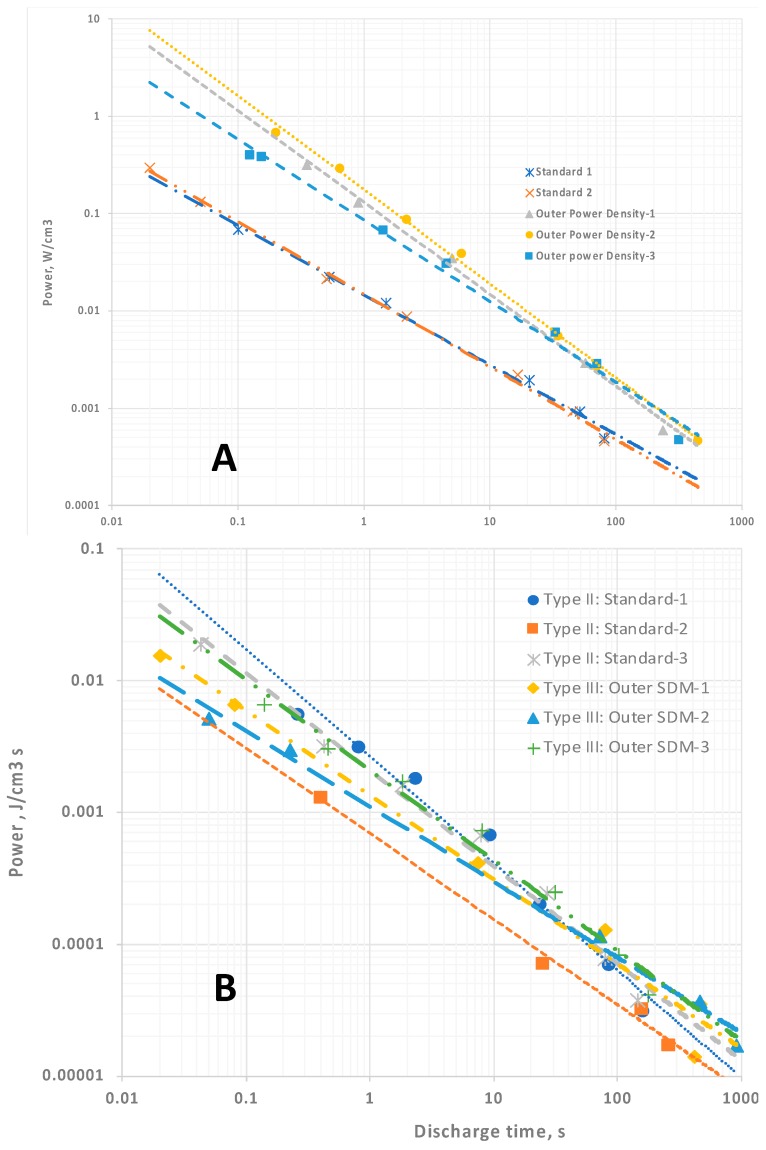
Power density. (**A**) Celgard-based capacitors. In all cases the power density increases, according to a simple power law relationship, as the discharge time decreases. (**B**) LLDPE-based capacitors. There is a nearly linear power law relationship for all data. The power density computed for Type III are arguably inflated because of the assumption that energy is only associated with the Type I: Core dielectric volume.

In contrast to dielectric constant, for the capacitors employing Celgard the trends for Type II capacitors in energy density (Figure 4A) and power density (Figure 5A) are well fitted as a function of discharge time with a simple power law. This reflects the method—the total area under the discharge curve is measured. Unlike the determination of the slope of the discharge curve, which is necessary for computation of dielectric constant, there is no “art” to making this measurement. This leads to cleaner, more reliable predictive values. It is straightforward and reliable. As discussed below, and argued elsewhere, energy and power density are better performance indicators than dielectric constant for NP supercapacitors.

For the capacitors employing LLDPE none of the data, that is, dielectric constants, energy (Figure 4B) and power density (Figure 5B), are perfectly described by a simple power law. The lack of precise fitting does not challenge SDM theory. The trends anticipated remain: dielectric constant and energy density trends monotonically downward with decreasing discharge time, and power density trends up monotonically as expected.

In sum, Type II: Standard NP supercapacitors behave as anticipated based on extrapolation of observations of other SDM based capacitors in terms of values, and trends with discharge time, of dielectric constant, energy and power density. One novel outcome from the present report is the demonstration that the use of metal electrodes does not change the fundamentals of SDM-based capacitors.

Type III—As shown in Figure 2, the discharge time for these capacitors is extremely long compared to Type I, a result clearly not anticipated by the standard model of dielectrics. The effective dielectric constants (EDC), computed assuming only the material between the electrodes contributes to this value, is greater than 10^7^ (Figure 3) for discharge times longer than ~10 s. Moreover, essentially the same results are obtained repeatedly for different Type III capacitors, validating the results.

The computation of enormous EDC values for Type III: Outer SDM is strong empirical support of the SDM hypothesis. That is, if the core is the only part of the structure of Type III capacitors that must be considered, why is the EDC as much as six orders of magnitude higher than that of Type I and Type IV capacitors? Not only is the computed EDC consistent with the SDM postulate, it is also a value range found only for NP supercapacitors. Indeed, ceramic capacitors have never been reported to have values higher than 10^5^. The energy (Figure 4) and power densities (Figure 5), computed based on only the Type I: Core dielectric volume, also attain values consistent with earlier reports of the unique high values found for several types of NP supercapacitors.

The results presented here for Type III capacitors disprove this standard model: Only the core section, that is, the Type I structure found at the center of Type III capacitors, is a significant part of the geometry. Indeed, if only the core part is significant, then the EDC for Type III capacitors would be similar to that of Type I (ca. 100). Given the measured value is repeatedly found to be as much as six orders of magnitude greater, the standard model is effectively debunked.

The high EDC values present a puzzle regarding how to present the results. The standard protocols, employed herein to compute values for Figure 4 and Figure 5, essentially to dramatize the impact of dielectric outside the core capacitor volume are not entirely satisfactory. Now that it is established that dielectric outside the core is contributing to the overall capacitor behavior, a new approach to identifying the “capacitor”, and concomitantly, the volume to be use in dielectric computation, must be developed. No proposed resolution is included in this manuscript.

At this juncture it is also appropriate to consider the relative magnitudes of Type II and Type III results. For Celgard-based capacitors, the “effective” dielectric constants, power and energy density values for Type II and Type III capacitors are in distinct regions of the plots (Figure 3A, Figure 4A and Figure 5A). The “magnitudes” do not overlap. In contrast, For the LLDPE based capacitors the Type II and Type III data fields clearly overlap’ (Figure 3B and Figure 4B,C). This is not a really meaningful result as the latter are effective values, and the former are computed without any need to consider the impact of dielectric material outside the capacitor core. For example, in terms of total energy there is far less energy in Type III capacitors of the same effective energy density than there is in a Type II capacitor of the same absolute energy density. This is because the volume associated with the computation of energy storage in the Type II system is far larger. This overlap does illustrate the difficulty of using the standard concepts to quantify capacitor properties, including energy and power density, in those cases for which dielectric material outside the core is having a pronounced influence. It also suggests “dielectric constant” is a meaningless, or even misleading, concept in these cases.

TYPE IV—Discharge data for a Type I capacitor shown in Figure 2, panels C and D, is very similar to that collected for Type IV capacitors. That is, the discharge behavior of these capacitor is very quick, no more than 10× slower than Type I capacitors with an identical core region. As discussed below this is consistent with a simple SDM model of the behavior observed for Type II capacitors. That is, ions must travel through the SDM material from the positive electrode to the negative electrode to effect the formation of a large dipole with polarity opposite that of the electrodes themselves. If this charge is blocked from travel, as in the case of Type IV capacitors, net high dielectric performance is not observed.

## 4. Discussion

All of the data are consistent with this first component of the SDM model: dielectrics increase the capacity,

(2)  C=q/V 

where *C* is capacitance, *q* is charge and *V* is volts, by partially cancelling the field created by charges on the capacitor electrodes at all points in space. In particular, the dielectric not only reduces the field between the electrodes, but also at all points in space outside the electrodes.

Given that the voltage is the integrated work done against the field,

(3) $$\;Voltage=\int_{0}^{\infty }\bar{E}\cdot dr\;$$

where *E* is the electric field, if the field at all points in space is reduced, the work (voltage) required to bring charges to the capacitor electrodes is reduced. Given that a dielectric reduces the field at all points in space, this naturally leads to an increase in capacitance, per Equation (1). In sum, it takes more stored electrons on the electrodes to reach a given voltage when a dielectric is present, because the net field at all points in space is lower when a dielectric is present.

In prior work the discussion of the effect of dielectric materials has been universally limited to the space between the electrodes. This ignores one of the fundamental tenants of field theory: voltage is a state property. Voltage is path independent, hence it does not matter if electrons reach the high voltage plate by passing through the dielectric or through space outside the dielectric from a zero-voltage location at infinite distance. Hence, if the dielectric reduces the field between the electrodes it must simultaneously be reducing the field at all points in space.

**Figure 6:**
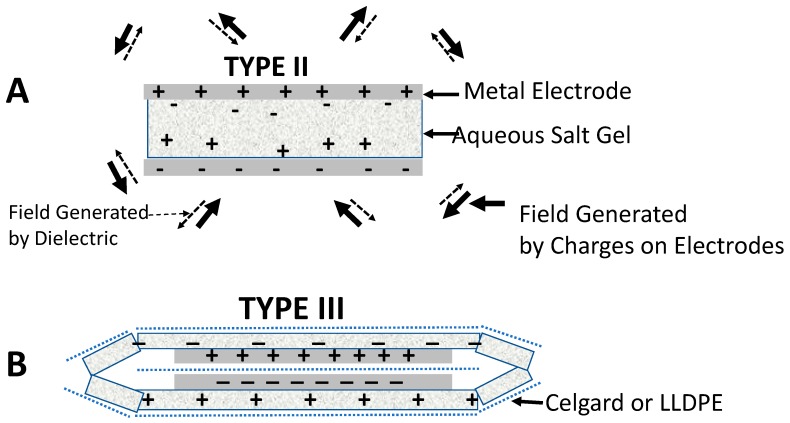
SDM hypothesis. (**A**) The dielectric material in a Type II capacitor is polarized such that the fields created by the dielectric (dashed arrows) are oppositely directed at all points in space to the fields created by the charges on the electrodes (solid arrows). (**B**) In a Type III: Outer SDM capacitor, charge travels between the two outer dielectric layers. This creates a dipole oppositely polarized to that created by charges on the electrodes. Hence, the net field at every point in space is lower relative to the condition in which no outer SDM dielectric layer is present. Thus, as per Equations (2) and (3), the capacitance is increased.

How does a dielectric reduce field strength according to SDM theory? The charges on the electrodes polarize the charges in a dielectric such that they produce fields opposite in direction to those produced by charges on the plates (Figure 6A). In the presence of a dielectric the field is reduced at every point in space relative to the case of no dielectric. Hence, for the same number of charges on the electrodes, the voltage at the plates is lower when a dielectric is present, per Equation (2). As discussed elsewhere, this means more charge must be brought to an electrode, by any path, to reach a target voltage for a capacitor with a dielectric than for one without a dielectric. More charge, that is, electrons or positive ions at a given voltage is equivalent to more stored energy at that voltage. Each stored electron has an associated energy.

A second key aspect of the SDM theory is that all dielectric behavior can be traced to the length of the dipoles formed in the dielectric. The larger the dipoles induced in a dielectric material by the field generated by the charges on the electrodes, the greater the net field reduction by the dielectric. As SDM dipoles form via ionic movement within a liquid media, the dipoles formed are far larger than those found in solids. As discussed in earlier reports, in SDM the dipole lengths are order of microns whereas in traditional solid dielectrics, such as barium titanate, the dipoles are a fraction of an Å in length. Clearly, this suggests that SDM materials should be far more effective at field reduction than any known solid dielectric. All observations, here and in past work, are clearly consistent with this predicted behavior.

It is also notable that the SDM model of field reduction is completely consistent with all classic models of static dielectric behavior. All classic models attribute dielectric properties to the strength of the dipoles induced by charge on the electrodes [1,7,24,25,26,27,28,29]. In the SDM model, the implicit in the classic models is merely made explicit. The dipoles impact performance by reducing the fields everywhere in space. In turn, more charge, (higher capacitance) is required to reach the same net electrode voltage.

The model is also consistent with classic frequency dependence models [27,30]. For ceramic capacitors, for which the dipoles are sub Å in length, it is well known there is no phase lag generally until the MHz or even Ghz frequency range is reached. Moreover, at that frequency at which the dipoles are no longer able to stay in alignment with the forcing field, there is an observable phase lag and capacitance roll off. A similar explanation can be applied to systems in which ions must physically travel, up to microns, through an electrolyte to form dipoles, such as in super dielectric materials, or in electric double layer capacitors (EDLC). At a conceptual level, the frequency at which roll off becomes significant should be a function of the dipole length. That is, the greater the distance charges must travel to form dipoles, the longer it takes. Also, it should be a function of the nature of the travel media. In an SDM the charges travel as ions in a viscous media. In both SDM and EDLC, the dipoles are far longer and the media far more viscous, than in solid dielectrics. This explains why for both SDM and EDLC a phase lag and/or capacitance decrease is generally notable even at 1 Hz, whereas in solids it is generally only observed in the MHz band. Also, the need to permit sufficient time for ionic travel to maximize charge separation distance, hence dipole strength and length, explains the long hold time at elevated voltage employed herein (see Experimental Methods), as well as in the standard characterization protocols employed with commercial EDLC capacitors.

The first component of the SDM model indicates any and all arrangements of dielectric material that result in the partial cancellation of the fields created by the charges on the electrodes will lead to improved dielectric values, energy density, etc. In this regard, Type III capacitors in this study are exemplary. As shown in Figure 6B, the charges on the electrodes will induce an opposite polarization of charges within the external dielectric material. In particular, charge in the form of ions, flows within the dielectric to produce a net positive dielectric material along the positive metal electrode and a net negative material along the negative metal electrode. This, in turn will reduce the net field at all points in space, including the volume between the electrodes. The net impact on parameters such as effective dielectric value, energy density, and power density should be similar to that observed for a conventional capacitor geometry (Type II).

The model requires that there must be a net charge transfer between dielectric material adjacent to the positive electrode and that adjacent to the negative electrode. In the absence of this charge transfer, the dipoles will form in each outer gel section and will be oppositely polarized to each other. Hypothesis: The electric fields of electrically isolated, outer dielectric layers will cancel. That is, the outer dielectrics of electrically isolated outer dielectric layers are oppositely polarized, and the electric fields created by the outer dielectric layers cancel at all points in space. In this case there should be little or no net effect to outer dielectrics. This is in fact observed for Type IV: Extended capacitor geometry. Type IV: Extended capacitors have a higher net dielectric value than Type I capacitors, but the measured effective dielectric values are three orders of magnitude below that required to earn the appellation SDM. A simple explanation for the difference between Type III and Type IV capacitors is presented. The extended dielectric of Type IV capacitors is a barrier to charge transfer between the gel material on the top and bottom of the capacitor. If no net charge can pass, no effective dipole can form.

Another conundrum associated with the present results is that Equation (1) for parallel plate capacitors, which heretofore has always been explicitly used to determine dielectric constant and implicitly to determine energy and power density, is not correct in those cases in which externally located dielectric material substantially reduces the field at all points in space. This leads to the conclusion that new language must be substituted for the current terms: dielectric constant, energy density and power density. In the present work the gap in terminology was (temporarily) filled by using the terms effective dielectric constant, etc.

Finally, it is instructive to dispel the notion that Type II and Type III capacitors, and in fact, all NP supercapacitors can be explained on the basis of EDLC theory. There are three experimental arguments presented on the basis of the present work. The first is based on the analogy between Type IV structure and EDLC structure. Specifically, in the event that the dielectric gel is a high surface area conductive material, a required characteristic of EDLC electrode material [1] the Type IV capacitors would be structurally equivalent to an EDLC/supercapacitor. That is, the gel would be the analog to high surface area carbon (e.g., graphene), the aqueous NaCl the electrolyte analog, and Celgard/LLDPE, the separator analog. Even one of the separator/extended dielectric materials employed herein, Celgard, is often used as the separator in EDLC. In sum, Type IV geometry is “supercapacitor” geometry. Hence, the finding that Type IV capacitors do not in any sense act as supercapacitors, dispels the notion that NP supercapacitors, including Type II and Type III, can be explained by standard EDLC models.

The second argument is based on Type III geometry. If, as required for the EDLC model, the fumed silica/aqueous NaCl gel is the analog to the high surface area conductive material of EDLC, then the gel, required to be highly conductive in the EDLC model, in Type III geometry would create a short between electrodes. Indeed, there is no separator between the electrodes in Type III geometry. The primary role of the separator in EDLC is to prevent shorting. Notably, there is no separator in any of the previous SDM studies [1,2,3,8,9,10,11,12,13,14,15,16], and no shorts were ever detected.

The third argument is a “reality” test. By employing a standard multimeter, it is clear that fumed silica/aqueous NaCl has a very, very low conductivity. 

## 5. Conclusions

The data presented here clearly illustrates that for a given geometry and charge/discharge cycle, dielectric material outside the volume generally referred to as “the capacitor” can increase the discharge time, and concomitantly energy storage, relative to the same capacitor minus all external dielectric. Specifically, it was observed that pasting a type of SDM gel around the outside of a standard parallel plate capacitor with a polymer dielectric increased discharge time by as much as six orders of magnitude. According to the standard paradigm, this is impossible for two reasons: (i) Material outside the region between the electrodes has no effect, and (ii) The effective dielectric constants measured, >100,000,000 in many cases, are larger than any observed by orders of magnitude, except in the case of SDM.

The scientific process is not employed to prove a theory, only to disprove one. On the basis of the above described results the standard, or text book, model of the mechanism of dielectric performance is debunked. Clearly the standard model, in which no consideration is given to the impact of any material outside the space between electrodes, cannot explain the observations reported. In contrast, the SDM model of dielectric behavior is found to be consistent in every respect with these novel results.

Finally, it is notable that the dielectric constant values reported for Type II capacitors, >10^8^ (see Table 1 and Table 2), represent the first report of SDM behavior for systems employing metal electrodes, rather than carbon electrodes.

## Figures and Tables

**Table 1:** Comparison of low current discharge times/effective dielectric constants for different types of Celgard and gel-based capacitors.

Gelgard and Gel Dielectric Capacitors			
Type-Version	Gel Thickness, Microns	Discharge Time, s (0.05 mA)	Dielectric Constant, <1 V, 0.05 mA
I	0	0.02(0.01 mA *)	75
II–1	325	125	5 × 10^8^
II–2	350	110	1.7 × 10^8^
III–1	355 **	70	2.3 × 10^7^
III–2	245	70	1.8 × 10^7^
III–3	220	57	1.9 × 10^7^
IV	110	0.11(0.01 mA)	6.2 × 10^2^

* For capacitor with very low dielectric values the discharge current was adjusted to permit measurement of time without voltage overshoot. ** Total gel thickness, top layer + bottom layer.

**Table 2:** Comparison of low current discharge times/effective dielectric constants for different types of LLDPE and gel-based capacitors.

LLDPE and Gel Dielectric Capacitors			
Type-Version	Gel Thickness, Microns	Discharge Time, s (0.05 mA)	Dielectric Constant, <1 V, 0.05 mA
I	0	0.004(0.025 mA *)	45
II–1	145	160	1.8 × 10^8^
II–2	85	156	1.6 × 10^8^
II–3	174	144	2.0 × 10^8^
III–1	102 **	460	8.4 × 10^7^
III–2	132	461	8.9 × 10^7^
III–3	266	175	2.9 × 10^7^
IV	110	0.04(0.025 mA *)	4.5 × 10^2^

* For capacitor with very low dielectric values the discharge current was adjusted to permit measurement of time without voltage overshoot. ** Total gel thickness, top layer + bottom layer.
